# Cooperative Interaction of Nck and Lck Orchestrates Optimal TCR Signaling

**DOI:** 10.3390/cells10040834

**Published:** 2021-04-07

**Authors:** Frederike A. Hartl, Jatuporn Ngoenkam, Esmeralda Beck-Garcia, Liz Cerqueira, Piyamaporn Wipa, Pussadee Paensuwan, Prapat Suriyaphol, Pankaj Mishra, Burkhart Schraven, Stefan Günther, Sutatip Pongcharoen, Wolfgang W. A. Schamel, Susana Minguet

**Affiliations:** 1 Faculty of Biology, University of Freiburg, 79104 Freiburg, Germany; frederike.hartl@biologie.uni-freiburg.de (F.A.H.); esmeralda.beck-garcia@roche.com (E.B.-G.); liz.cerqueira@yahoo.com (L.C.); wolfgang.schamel@biologie.uni-freiburg.de (W.W.A.S.); 2 Signalling Research Centres BIOSS and CIBSS, University of Freiburg, 79104 Freiburg, Germany; 3 Center of Chronic Immunodeficiency CCI, University Clinics and Medical Faculty, 79106 Freiburg, Germany; 4 Department of Microbiology and Parasitology, Faculty of Medical Science, Naresuan University, Phitsanulok 65000, Thailand; jatupornn@nu.ac.th (J.N.); piyamaporn03@gmail.com (P.W.); 5 Department of Optometry, Faculty of Allied Health Sciences, Naresuan University, Phitsanulok 65000, Thailand; pussadeep@nu.ac.th; 6 Division of Bioinformatics and Data Management for Research, Research Group and Research Network Division, Faculty of Medicine Siriraj Hospital, Mahidol University, Bangkok 10700, Thailand; prapatsuriyaphol@gmail.com; 7 Pharmaceutical Bioinformatics, Institute of Pharmaceutical Sciences, University of Freiburg, 79104 Freiburg, Germany; pankaj.mishra@pharmazie.uni-freiburg.de (P.M.); stefan.guenther@pharmazie.uni-freiburg.de (S.G.); 8 Institute of Molecular and Clinical Immunology and Health Campus Immunology, Infectiology and Inflammation, Medical Faculty, Otto-von-Guericke University Magdeburg, 39120 Magdeburg, Germany; burkhart.schraven@med.ovgu.de; 9 Division of Immunology, Department of Medicine, Faculty of Medicine, Naresuan University, Phitsanulok 65000, Thailand; sutatipp@nu.ac.th; 10 Center of Excellence in Petroleum, Petrochemical, and Advanced Materials, Faculty of Science, Naresuan University, Phitsanulok 65000, Thailand

**Keywords:** TCR, conformation, Lck, Nck, RK motif

## Abstract

The T cell antigen receptor (TCR) is expressed on T cells, which orchestrate adaptive immune responses. It is composed of the ligand-binding clonotypic TCRαβ heterodimer and the non-covalently bound invariant signal-transducing CD3 complex. Among the CD3 subunits, the CD3ε cytoplasmic tail contains binding motifs for the Src family kinase, Lck, and the adaptor protein, Nck. Lck binds to a receptor kinase (RK) motif and Nck binds to a proline-rich sequence (PRS). Both motifs only become accessible upon ligand binding to the TCR and facilitate the recruitment of Lck and Nck independently of phosphorylation of the TCR. Mutations in each of these motifs cause defects in TCR signaling and T cell activation. Here, we investigated the role of Nck in proximal TCR signaling by silencing both Nck isoforms, Nck1 and Nck2. In the absence of Nck, TCR phosphorylation, ZAP70 recruitment, and ZAP70 phosphorylation was impaired. Mechanistically, this is explained by loss of Lck recruitment to the stimulated TCR in cells lacking Nck. Hence, our data uncover a previously unknown cooperative interaction between Lck and Nck to promote optimal TCR signaling.

## 1. Introduction

The T cell antigen receptor (TCR) is a multimeric transmembrane protein, exclusively expressed on the surface of T cells. It is composed of the ligand-binding clonotypic TCRαβ heterodimer and the noncovalently associated invariant CD3 signal-transducing complex [1]. This CD3 complex includes the CD3εγ, CD3εδ, and ζζ signal transduction chains [2]. Each CD3 subunit has a single cytoplasmic immunoreceptor tyrosine-based activation motif (ITAM) except the ζ cytoplasmic tail which has three ITAMs [3]. ITAMs are crucial to transduce activation signals into the T cell. Although a complete structure of the TCR is still unknown, accumulating evidence suggests that the TCR switches between different conformational states [4,5,6,7]. Binding of the TCR’s ligand (major histocompatibility complex bound to an antigenic peptide; pMHC) selects and stabilizes the TCR in active conformations that were hardly visited in the absence of ligand binding (reviewed in) [8,9]. Only the active conformations support the transmission of activation signal from the extracellular to the intracellular domains of the TCR [10,11,12].

Intracellular signaling is initiated by the phosphorylation of the ITAM tyrosines by the lymphocyte-specific kinase (Lck) [13]. Phosphorylated ITAMs serve as docking sites for the two Src homology 2 (SH2) domains of the ζ chain-associated protein of 70 kDa (ZAP70), which is then phosphorylated by Lck to unleash ZAP70’s full catalytic activity [14,15]. ZAP70 then phosphorylates a number of signaling proteins, including the linker for the activation of T cells (LAT) and the SH2-domain-containing leukocyte protein of 76 kDa (SLP76), leading to signal progression. Once an activation signal is initiated, several mechanisms contribute to intensify or sustain TCR signals. These mechanisms include ligand-induced receptor clustering, recruitment of the co-receptors CD4 and CD8, or the segregation of the TCR from phosphatases (reviewed in) [16].

Various reports have demonstrated that the CD3ε chain has a special role in TCR signaling after ligand binding. The CD3ε cytoplasmic tail contains a number of unique interaction motifs that are crucial for TCR signaling (reviewed in) [17]. These motifs include (from the N- to C-terminus) a basic amino acid rich sequence (BRS), a proline-rich sequence (PRS) and a receptor kinase (RK) motif. Ionic interactions of the BRS with acid residues in the unique domain of Lck are known to contribute to TCR signaling [18]. In addition, interactions of the BRS with p85 promote PI3K/AKT signaling [19]. The PRS, which is only accessible in the active TCR conformations, serves as a docking site for the adaptor protein non-catalytic region of tyrosine kinase (Nck) [4]. Out of the three SH3 domains and one SH2 domain of Nck, the SH3.1 domain binds to the CD3ε PRS [4]. Mice expressing a CD3ε with a mutation in the PRS fail to recruit Nck to the TCR, and show impaired ζ phosphorylation, reduced ZAP70 recruitment to the TCR, and consequently, reduced ZAP70 phosphorylation [20]. Likewise, down-regulation of Nck expression in human T cell lines impaired TCR-induced phosphorylation of the kinases ERK and MEK, and subsequently reduced IL-2 and CD69 expression upon TCR stimulation [21]. The RK motif has been recently identified as a new motif recruiting Lck to the ligand-bound TCR [22]. Like the PRS [4] and the ITAM [10] of CD3ε, the RK motif is only exposed in the active TCR conformation [22]. The discovery of the RK motif supports a new model of how phosphorylation of the TCR is initiated: ligand binding stabilizes the active TCR conformations, resulting in the exposure of the RK motif and the ITAMs; Lck binds with its SH3 domain to the RK motif, recruiting Lck to the TCR to phosphorylate the ITAMs. Recruitment of the CD4- or CD8-bound Lck might further enhance and/or stabilize TCR signaling [23,24,25,26].

Interestingly, double mutation of the PRS and the RK motif in T cell lines showed a profounder TCR signaling defect compared to single mutations of either the PRS or the RK motif alone [22]. Likewise, mice and T cell lines modified to prevent the TCR from switching to the active conformation, in which neither the PRS nor the RK motif are exposed, exhibit an almost complete block in TCR function [27]. These results suggest that both Nck and Lck recruitment might contribute to fostering TCR signaling. The cooperation of Nck and Lck at the TCR might be more complex than anticipated as uncovered by the existence of contradicting results in the literature. On the one hand, it has been shown that Nck and Lck interact with each other through an adapter protein called T cell-specific adaptor protein (TSAd) [28]. On the other hand, TSAd-independent binding of Nck to Lck has also been observed [29]. 

Here, we tested the hypothesis that recruitment of Nck and Lck to CD3ε promotes proximal TCR signaling. Indeed, we could show that Nck silencing in Jurkat T cells led to reduced phosphorylation of the TCR and ZAP70, resulting in a decrease in ZAP70 recruitment to the TCR. Reduced TCR signaling was a consequence of a loss of Lck recruitment to the TCR in the absence of Nck. Molecular modeling demonstrated that simultaneous binding of the SH3.1 domain of Nck (Nck(SH3.1)) and the SH3 domain of Lck (Lck(SH3)) to the same CD3ε molecule is possible, fostering the idea that the recruitment of these molecules to the TCR precedes and regulates ITAM phosphorylation upon ligand binding. Finally, using an in vitro kinase assay, we show that Lck and Nck cooperate for optimal CD3 phosphorylation. 

## 2. Materials and Methods

### 2.1. Cell Lines

31–13.scTCRβ cells were already described. Briefly, TCRβ- deficient human Jurkat cell line was stably transfected to express a TCRβ containing a nitro-iodo-phenol (NIP)-specific single chain Fv fragment (scFv) coupled to the TCRβ chain. In 31–13 scTCRβ cells, TCRs can be purified through the NP-specific scFv without altering the TCR conformation [5]. Silencing of Nck1 or Nck2 or both Nck isoforms by specific short hairpin RNA (shRNA) in Jukat T cells (E6–1) was previously described [21]. Cells were maintained in RPMI 1640 medium (Gibco) supplemented with 10% heat-inactivated fetal bovine serum (FBS) (Gibco), 2 mM L-glutamine and 100 U/mL penicillin and 100 µg/mL streptomycin (HyClone, Fisher) in a humidified incubator with 5% CO_2_ at 37 °C.

### 2.2. Antibodies and Chemicals

The following antibodies were used: anti-Nck1, anti-ZAP70, anti-Lck, anti-phospho-Lck (Src416) and anti-phospho-ZAP70 (Y319, Cell Signaling Technology), anti-phospho-ζ (Y142, Sigma-Aldrich), anti-idiotypic TCR (C305, Millipore), anti-CD3ε (M20, Santa Cruz Biotechnology), anti-CD3ε (OKT3, eBioscience), anti-ζ antiserum 448 [4], anti-phospho-CD3ε (Y188) [30], anti-GST (Bethly), anti-GAPDH (Sigma) and secondary antibodies for immunoblotting (Perbio). Alexa Fluor 647-labeled anti-CD3ε (UCHT1, BioLegend) was used for flow cytometry. Recombinant His-tagged Nck was from Sigma and recombinant active Lck (aa61-aa509) was a generous gift from B.F. Lillemeier, Salk Institute for Biological Studies, San Diego.

### 2.3. In Situ Proximity Ligation Assay (PLA)

PLA was performed as previously described [22,31,32]. Briefly, cells were starved and harvested on diagnostic microscopic slides (Thermo Scientific) at 37 °C for at least 1 h. Cells were either left unstimulated as a control or stimulated with anti-TCR (C305, 1:50) or 10 μg/mL anti-CD3ε (OKT3) at 37 °C for 5 min, followed by a 15 min fixation step with 2% paraformaldehyde. Cells were permeabilized using 0.5% saponin and blocked for 30 min. Cells were then stained with antibody pairs between the goat anti-CD3ε (M20) or anti-CD3δ (M20), both from Santa Cruz, and either a rabbit anti-ZAP70 or a rabbit anti-Lck antibody (both Cell Signaling Technology) with an incubation overnight at 4 °C. A proximity assay between CD3ε and ZAP70, and CD3δ and Lck molecules, shown by red fluorescence signal dots, was carried out using the Duolink kit (Olink Bioscience) according to the manufacturer’s instructions. Nuclei were stained with DAPI (Roth). Images were taken with a fluorescence microscope (Nikon Eclipse Ti and Nikon C2) and analyzed with the Blob-Finder program (Uppsala University).

### 2.4. Cell Stimulation and Lysis for Biochemistry Analysis

5 × 10^6^–15 × 10^6^ cells per sample were resuspended in serum-free RPMI medium and incubated for 1 h at 37 °C. Cells were left unstimulated or stimulated with 1–5 μg/mL of anti-CD3ε (OKT3) antibody at 37 °C at the indicated time points. After stimulation, cells were lysed for 20 min on ice in 0.5–1 mL lysis buffer containing 20 mM Tris-HCl (pH 8), 137 mM NaCl, 2 mM EDTA, 10% glycerol, protease inhibitor cocktail (Sigma), 1 mM PMSF, 5 mM iodoacetamide, 0.5 mM sodium orthovanadate, 1 mM NaF and 0.3% Brij96V. Lysis was followed by a 15 min centrifugation to pellet the nuclei and insoluble materials. The supernatants were subsequently used as indicated.

### 2.5. In Vitro Kinase Assay

30 × 10^6^ 31–13.scTCRβ cells [5] were incubated with the kinase inhibitor PP2 (10 µM) for 1 h. Next, cells were left untreated or treated with 10 µg/mL anti-CD3ε (OKT3) antibody for 1 h on ice to stabilize the active TCR conformation. Upon lysis, scTCRs (and bound antibodies) were purified with nitro-phenol coupled-sepharose beads. After washing, purified TCRs were subjected to an in vitro kinase assay. To this end, 45 nM active, recombinant Lck alone, 45 nM recombinant His-tagged Nck (Sigma) alone or both together and 0.4 mM ATP were added in kinase-buffer (40 mM HEPES, 10 mM MgCl_2_, 2 mM MnCl_2_) and incubated for 15 min at 30 °C. Reaction was stopped by adding sample buffer followed by boiling for 5 min.

### 2.6. Pull-Down (PD) Assay, Immunoprecipitation (IP) and Immunoblotting 

GST-Nck(SH3.1) and GST-Lck(SH3) were expressed in the *E. coli* strain BL21, coupled to glutathione sepharose (GE Healthcare) and incubated with lysates as described [22]. For TCR immunoprecipitation (TCR-IP), 2 μg of anti-CD3ε (OKT3) antibody together with 10 μL protein G coupled sepharose beads (GE Healthcare) were added to lysates. PD assay and TCR-IP were performed by overnight incubation at 4 °C.

Proteins from lysate, PD or TCR-IP were subjected to SDS-PAGE followed by immunoblotting according to standard procedures. Protein bands were detected by chemiluminescence under a CCD camera (ImageQuant LAS 4000; GE Healthcare). Relative band intensity was quantified by ImageJ software and ImageQuantTL software (GE Healthcare).

### 2.7. Molecular Modeling

Molecular modeling was performed as described in the results section using the HADDOCK (High Ambiguity Driven biomolecular DOCKing) web server (version 2.2) [33] to simulate the docking of the proteins Lck(SH3), Nck(SH3.1) and CD3ε. Homology modeling was performed using MODELLER (v.9.13) [34] and a default ‘DOPE’ score was chosen to select the best human CD3ε model as specified in the result.

### 2.8. Quantification and Statistical Analysis

Data are represented as means ± SEM. Statistical significance was calculated by Student’s *t*-test using GraphPad PRISM 6. Differences were considered significant when the *p* values were <0.05. 

## 3. Results

### 3.1. Impaired Proximal TCR Signaling in the Absence of Nck

The importance of Nck on distal TCR signaling was previously demonstrated, as silencing of Nck1 and Nck2 resulted in the reduction of CD69 up-regulation and IL-2 secretion upon TCR stimulation [21]. Therefore, we aimed to investigate whether Nck also regulates early proximal signaling events upon TCR triggering. One of these events is the phosphorylation of ZAP70 on tyrosine 319 (Y319) by the Src family kinase Lck, which is involved in ZAP70 activation [35] and serves as a docking site for other molecules controlling downstream signaling (reviewed in) [36]. In mouse models, mutations in the CD3ε PRS prevent recruitment of Nck to the TCR resulting in impaired ZAP70 recruitment and phosphorylation, which suggests that the early recruitment of Nck to the TCR modulates ZAP70 activation [20].

**Figure 1:**
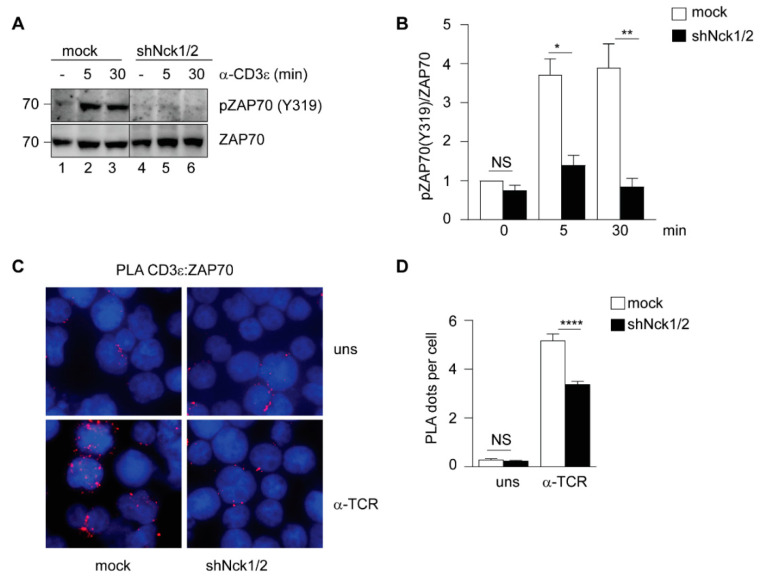
Nck is needed for ZAP70 phosphorylation and recruitment to the activated TCR. (**A**) Mock-treated or shNck1/2 knock-down Jurkat cells were left unstimulated (-) or stimulated with 1 μg/mL anti-CD3ε antibody (OKT3) for the indicated times at 37 °C. Total cell lysates were subjected to immunoblot with anti-phospho-ZAP70 (Y319) and anti-ZAP70 antibodies. (**B**) Data from three independent experiments as shown in A were quantified and unpaired Student’s *t*-test was performed between the indicated samples. (**C**) In situ PLA between the TCR (CD3ε) and ZAP70 showing a TCR-ZAP70 distance less than 80 nm (red fluorescent signal) in mock-treated or in shNck1/2 knock-down Jurkat cells. Cells were either left unstimulated (uns) or stimulated with anti-TCR (C305) at 37 °C for 5 min. Nuclei were stained with DAPI. One representative experiment is shown. (**D**) Quantification and statistical analysis of three independent experiments of data shown in C using unpaired Student’s *t*-test. Mean values  ±  SEM are shown. * *p* < 0.05, ** *p* < 0.01, **** *p* < 0.0001, NS, not significant.

To investigate ZAP70 phosphorylation upon TCR stimulation, we created Jurkat T cells either mock-treated or shRNA-treated to silence both Nck1 and Nck2 (named shNck1/2) (Supplementary Figure S1A,B). Importantly, absence of both, Nck1 and Nck2, did not affect surface TCR expression (Supplementary Figure S1B). Upon TCR stimulation with an anti-CD3ε antibody, mock-treated cells showed an increase in the phosphorylation of ZAP70 on Y319 as expected. However, TCR stimulation did not lead to an increase in ZAP70 phosphorylation in shNck1/2 cells (Figure 1A,B). 

To test whether absence of ZAP70 phosphorylation was the result of a defect in ZAP70 recruitment to the TCR, we performed an in situ proximity ligation assay (PLA) between the TCR and ZAP70. In this assay, the presence of a red dot indicates proximity between the TCR and ZAP70. The specificity of this approach is demonstrated (Supplementary Figure S1C). In resting cells, hardly any ZAP70 was found in the vicinity of the TCR. Upon TCR stimulation with an anti-CD3 antibody, ZAP70 was recruited close to the TCR in mock-treated cells as indicated by the detection of red dots. In contrast, ZAP70 recruitment to the TCR after TCR stimulation was severely reduced in shNck1/2 cells compared to mock-treated cells (Figure 1C,D). These results are in line with a previous report demonstrating that Jurkat cells expressing a mutant Nck1, that was unable to bind to the TCR, also failed to recruit ZAP70 to the stimulated TCR [31].

**Figure 2:**
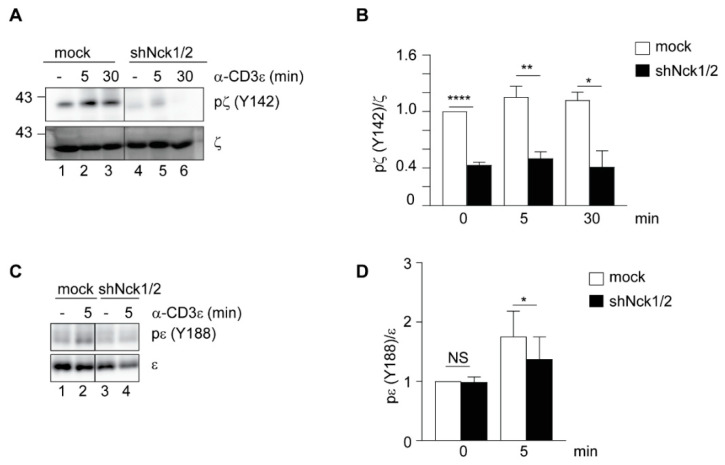
TCR phosphorylation depends on the presence of Nck. (**A**) Cells were left unstimulated (-) or stimulated with 1 μg/mL anti-CD3ε antibody (OKT3) at the indicated times at 37 °C. Total cell lysates were subjected to immunoblot with anti-phospho-ζ (Y142) and anti-ζ antibodies. (**B**) Data from three independent experiments performed as in A were pooled and unpaired Student’s *t*-test was performed between the indicated samples. (**C**) Cells were stimulated as in A. Total cell lysates were subjected to TCR-immunoprecipitation and immunoblotting with anti-phospho-ε (Y188) and anti-ε antibodies. (**D**) Data from three independent experiments performed as in C were pooled and paired Student’s *t*-test was performed between the indicated samples. Mean values  ±  SEM are shown. * *p* < 0.05, ** *p* < 0.01, **** *p* < 0.0001, NS, not significant.

Tyrosine-phosphorylated ITAMs of the CD3 chains serve as docking sites for ZAP70 [13]. Therefore, we next investigated whether reduced ZAP70 recruitment to the TCR is a consequence of impaired ITAM phosphorylation. While mock-treated cells showed an increase in ζ phosphorylation after TCR stimulation, hardly any ζ phosphorylation was detected in shNck1/2 cells (Figure 2A,B). Similar results were obtained for CD3ε phosphorylation (Figure 2C,D). Altogether, these data support the hypothesis that Nck is required for proximal early TCR signaling, namely for optimal ITAM phosphorylation, ZAP70 recruitment to the TCR and subsequent ZAP70 phosphorylation. 

### 3.2. Nck Is Required for Optimal Lck Binding to the TCR

Next, we investigated whether Lck binding to the TCR is affected by the absence of Nck. Lck can be recruited to the phosphorylated ITAMs via its SH2 domain [37]. However, prior its binding via the SH2 domain, Lck needs to gain access to the ITAMs to phosphorylate them. We have recently solved this apparent paradox by demonstrating that Lck is first recruited to the unphosphorylated TCR via the interaction of Lck(SH3) with the RK motif that is located in the CD3ε cytoplasmic tail. Importantly, the RK motif is exposed only upon ligand binding to the TCR, explaining how Lck discriminates between ligand-bound and resting TCRs [22]. 

**Figure 3:**
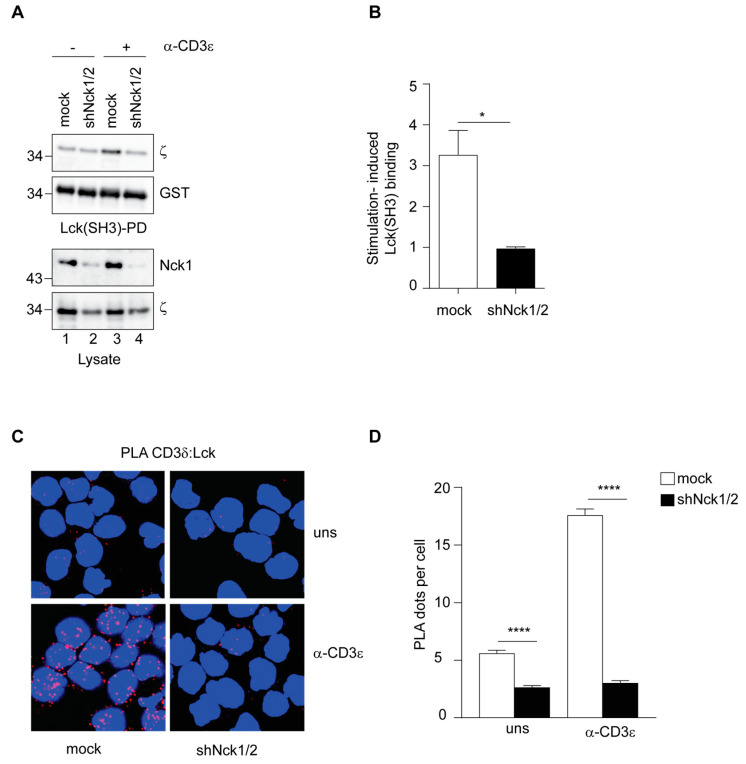
Nck facilitates Lck binding to the TCR. (**A**) The total cell lysate of unstimulated (-) or anti-CD3ε (OKT3) antibody stimulated (+) mock-treated and shNck1/2 Jurkat cells was subjected to a pull-down assay with GST-Lck(SH3)-coupled beads followed by immunoblotting with the indicated antibodies. Lysates served as control to show Nck1/2 downregulation. One representative experiment is shown. (**B**) The stimulation-induced binding of the TCR to Lck(SH3)-beads was calculated from three independent experiments performed as in A and analyzed using an unpaired Student’s *t*-test. (**C**) In situ PLA between the TCR (CD3δ) and Lck was performed. Jurkat mock-treated or shNck1/2 cells were either left unstimulated (uns) or stimulated for 5 min at 37 °C with 10 µg/mL anti-CD3ε antibody (OKT3). One representative experiment out of three is shown. (**D**) Quantification of the experiment shown in C and analysis using an unpaired Student’s *t*-test. Mean values  ±  SEM are shown. * *p* < 0.05, **** *p* < 0.0001.

Firstly, we quantified the binding of the TCR complex to Lck(SH3)-coated beads using a pull-down assay followed by Western blot to detect ζ. Of note, ζ is the last subunit that is incorporated to the TCR complex in the endoplasmic reticulum and therefore, it is broadly used to detect the fully assembled TCR-CD3 complex [4,5,10,22]. As expected, Lck binding to the TCR was increased in antibody-stimulated mock cells compared to resting cells (Figure 3A,B). In contrast, stimulation of the TCR failed to induce TCR-Lck(SH3) binding in shNck1/2 cells (Figure 3A,B). We performed then a pull-down assay using the SH3.1 domain of Nck and observed that stimulation of the TCR did not result in TCR-Nck(SH3.1) binding in cells lacking Nck (Supplementary Figure S2A,B). These results suggest that endogenous Nck might help to stabilize the open TCR conformation and thereby, facilitate the exposure of the RK motif for Lck recruitment, of the PRS for Nck binding, and of the ITAM of CD3ε for subsequent phosphorylation by Lck [4,10,22].

Next, we investigated whether binding of endogenous Lck to the TCR was also affected in cells lacking Nck. To this end, we performed a PLA between the TCR and Lck, either in mock-treated or in shNck1/2 cells. Technical controls confirmed the specificity of this approach (Supplementary Figure S2C). As previously described [22,32], we observed a low level of Lck-TCR proximity in unstimulated mock cells (Figure 3C,D). Interestingly, this basal proximity between Lck and TCR was significantly reduced in the absence of Nck (Figure 3C,D). This observation supports the idea that Nck regulates the proportion of TCRs that are in the active conformations even in resting T cells [10]. In mock cells, TCR stimulation increased the proximity between the TCR and Lck as a result of multiple interactions via the SH3 and the SH2 domains of Lck. In contrast, stimulation in the absence of Nck failed to increase the recruitment of Lck to the TCR (Figure 3C,D). These data are in line with the observed reduction of Lck(SH3) binding to the TCR (Figure 3A,B) and of the absence of phosphorylation of the CD3 ITAMs observed in shNck1/2 cells (Figure 2). 

Altogether, our results demonstrate that Nck is needed for optimal binding of Lck to the TCR, and explains the strong reduction of both Lck-mediated ITAM phosphorylation and of TCR downstream signaling in cells lacking Nck. 

### 3.3. Simultaneous Binding of Nck and Lck to the TCR Orchestrates Optimal TCR Phosphorylation

**Figure 4:**
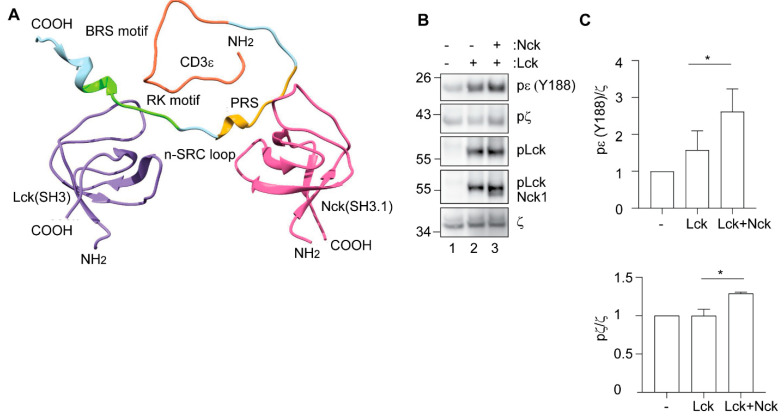
Simultaneous binding of Nck and Lck to the TCR enhances CD3 phosphorylation. (**A**) Backbone representation of the modeled complex formed by Lck(SH3) and Nck(SH3.1) with the CD3ε cytoplasmic tail. Purple, Lck(SH3); pink, Nck(SH3.1); multicolor, CD3ε. CD3ε motifs are depicted. Orange, Basic Rich Sequence, BRS; yellow, Proline-Rich Sequence, PRS; green, Receptor Kinase, RK motif. (**B**) 31–13.scTCRβ cells are TCRβ-deficient Jurkat cells expressing an scTCRβ composed of the nitro-iodo-phenol (NIP)-binding variable immunoglobulin domains of a NIP-specific antibody connected by a flexible linker to the N-terminus of wild-type TCRβ. 31–13.scTCRβ cells were incubated with anti-CD3ε (OKT3) antibody on ice for 1 h in the presence of PP2 and the TCR was purified using NP-coupled beads. An in vitro kinase assay was performed in the presence of the indicated proteins followed by immunoblotting. ATP was added to all samples. One representative experiment is shown. (**C**) Quantification of three experiments performed as in B is shown. Analysis was done using a paired Student’s *t*-test. Mean values  ±  SEM are shown. * *p* < 0.05.

As the RK motif and the PRS are located close to each other in CD3ε, we were interested to know whether Nck and Lck can bind simultaneously to the same CD3ε chain. To address this, HADDOCK (High Ambiguity Driven biomolecular DOCKing) web server was used to simulate the docking of Lck(SH3) and Nck(SH3.1) to CD3ε. We utilized the three-dimensional apo structures of human Lck(SH3) (Protein Data Bank entry 2IIM) and human Nck(SH3.1) (Protein Data Bank entry 2JS2). Since the structure of human CD3ε is unavailable, a structural homology model was generated using the mouse CD3ε structure as a template (Protein Data Bank entry 2K4F; sequence identity-90.91%). Homology modeling was performed using MODELLER (version 9.13) [34] and default ‘DOPE’ score was chosen to select the best human CD3ε model. HADDOCK allows for the integration of a wide range of experimental data including the chemical shift perturbations and site directed mutagenesis as ambiguous interaction restraints (AIRs). The information for the interacting residues as AIRs for Nck(SH3.1)-CD3ε complex was collected from literature [38,39]. The interacting amino acids as AIRs for the Lck(SH3)-CD3ε complex were previously described by us [22]. The top structure, having the lowest total energy, based on highest cluster size and most negative z-score, was selected as the final model (Figure 4A). The results suggest that the binding of one SH3 domain (from Nck or Lck) does not sterically hinder the binding of the second SH3 domain (from Lck or Nck, respectively) to the same CD3ε molecule. Thus, simultaneous binding of both molecules, Lck and Nck, to the same CD3ε is, at least theoretically, possible. 

We next tested whether simultaneous binding of Lck and Nck to CD3ε can promote TCR phosphorylation. To exclude conformational change of the TCR during the TCR purification step, we used 31–13.scTCRβ cells. These cells stably express a TCRβ chain coupled via a flexible linker to a nito-iodo-phenol (NIP)-specific single chain variable fragment (scFv). The scTCRβ is assembled within the TCR-CD3 complex and allows its purification [5]. We first prevented TCR phosphorylation by incubating 31–13.scTCRβ cells with the Src kinase inhibitor PP2, and thereby avoided the co-purification of any endogenous signaling protein including Lck and Nck. Next, we incubated the cells with antibodies that stabilize the active TCR conformation. The TCRs in the active conformation were then purified using the NIP-specific scFv and incubated either without Lck, with constitutively active recombinant Lck or with both, recombinant Lck and Nck and an in vitro kinase assay was performed (Figure 4B,C). Constitutively, active Lck was detected by phosphorylation of its kinase domain (Figure 4B and Supplementary Figure S3, pLck). The TCR was only phosphorylated in the presence of active Lck validating the specificity of our assay (Figure 4B,C and Supplementary Figure S3). When the in vitro kinase assay was performed in the presence of both Lck and Nck, the phosphorylation of CD3ε and ζ ITAMs was significantly increased compared to Lck alone (Figure 4B,C). Hence, these results suggest that Lck and Nck cooperate for optimal TCR phosphorylation. 

## 4. Discussion

Ligand binding to the TCR stabilizes the receptor in an active conformation exposing the CD3ε cytoplasmic tail for interaction with signaling proteins (reviewed in) [8,9]. The cytoplasmic tail of CD3ε contains motifs mediating protein–protein interactions prior to phosphorylation. These motifs are thus appealing candidates for the initiation of TCR signaling. Among these motifs, the PRS binds to the SH3.1 domain of Nck (Nck(SH3.1)) and thereby recruits Nck to the TCR independent of phosphorylation [4]. In addition, we have recently identified the RK motif, a non-canonical binding motif for the SH3 domain of Lck (Lck(SH3)). This motif is responsible for the recruitment of Lck to the TCR prior to ITAM phosphorylation [22]. The importance of Lck for TCR signaling has already been demonstrated [40,41,42,43,44]. However, Lck is a complex modular protein whose regulation at the TCR remains to be fully understood. Catalytic activity of Lck is regulated by at least two cooperative intramolecular interactions: (1) between the SH3 domain and a proline-rich sequence situated in the linker connecting the SH2 and kinase domains, and (2) between the SH2 domain and the phosphorylated Y505 in the C-terminal tail. The auto-inhibited Lck is a sensitive mousetrap that can be activated by alternative interactions [45]. For instance, SH3 domain displacement via binding to PxxP sequences or via the RK motif increases the local activity of Lck [22,46,47,48,49]. Auto-phosphorylation of Y394 is the strongest activator of Lck catalytic activity. In this study, we shed new light on the interplay between Lck and Nck at the TCR, showing that they cooperate to phosphorylate the ITAMs and thereby to recruit ZAP70 to the TCR.

The proximity of the PRS and the RK motif in the CD3ε cytoplasmic tail made us wonder whether both SH3 domains (from Nck and Lck) are able to bind to the same CD3ε molecule simultaneously. Alternatively, Nck and Lck could be recruited to two different CD3ε molecules of the same TCR. Using molecular modeling, we demonstrated that simultaneous binding of Nck(SH3.1) and Lck(SH3) to the same CD3ε chain is possible. This model provides support for the hypothesis that CD3ε bridges Nck and Lck at the TCR, and that these two molecules coordinate to regulate the phosphorylation of the ITAMs, and thereby, proximal TCR signaling. This idea is further supported by our experiments using T cells with either a single mutation in the RK motif or in the PRS of CD3ε, as well as simultaneous mutations of both motifs. Mutation of the RK motif reduced TCR signaling, including ITAM phosphorylation and T cell activation [22]. Mutation of the PRS in CD3ε also caused reduced ITAM phosphorylation and T cell activation in primary murine cells [20]. These results were reproduced using Jurkat-derived T cell lines alongside cells expressing CD3ε with both mutations, in RK motif and PRS. The double mutants displayed much stronger defects in TCR signaling compared to the single individual mutations [22]. The reduction in TCR phosphorylation and T cell activation in cells expressing the double mutations was comparable to cells expressing a CD3ε variant that cannot stabilize the active conformation, and therefore cannot expose the motifs to recruit Lck nor Nck to the TCR [22]. These data suggest that Nck might be required to promote proximal TCR signaling. However, mutations in the PRS might have other biological effects beyond the recruitment of Nck. In this study, we demonstrated that Nck promotes proximal TCR signaling upon ligand binding. Using biochemical approaches and proximity ligation assay (PLA), we showed that Nck is required for optimal TCR phosphorylation, ZAP70 phosphorylation by Lck, and ZAP70 recruitment to the TCR. Mechanistic insight came from our finding that Lck was not in proximity to the TCR upon stimulation in the absence of Nck. These data strongly suggest that Nck is needed for efficient Lck recruitment to the ligand-bound TCR to phosphorylate the ITAMs. At least two, not mutually exclusive, scenarios might explain these results: (1) Nck interacts, directly or indirectly, with Lck to enforce the binding of Lck to the ligand-bound TCR and (2) Nck stabilizes the active TCR conformation promoting the exposure of the RK motif and thereby the recruitment of Lck to the ligand-bound TCR. Regarding the first scenario, previous reports have suggested interactions between Nck and Lck [17,20,28]. An indirect interaction of Lck and Nck, through the adaptor protein TSAd, has already been reported [28]. In addition, Lck and Nck might interact with each other in a TSAd-independent manner, either by directly binding to each other or through an unknown interaction partner [28]. Supporting the second scenario are our results of pull-down assays with purified SH3 domains. In contrast to mock cells, the stimulated TCR failed to bind to either the Lck(SH3)- or the Nck(SH3.1)-coated beads in cells lacking Nck expression. This observation strongly suggests that endogenous Nck might help to stabilize the active, open TCR conformation, and thereby facilitate the exposure of the RK motif for Lck recruitment and the ITAMs for subsequent phosphorylation by Lck. This second scenario is also compatible with direct or indirect interactions between Lck and Nck.

Nevertheless, the idea of interplay between Nck and Lck to promote ITAM phosphorylation was additionally supported by our in vitro kinase assays. Adding recombinant Nck to an Lck in vitro kinase assay significantly enhanced ITAM phosphorylation compared to Lck alone. Thus, Nck and Lck seem to cooperate for TCR phosphorylation in the absence of additional proteins such as TSAd, as demonstrated in our minimalistic in vitro kinase assay. These results are in line with the notion of Nck supporting proximity between Lck and the ligand-bound TCR as discussed above. Nck might also increase the local activity of Lck at the TCR, for example, by providing alternative interactions (direct or indirect, see above) to the endogenous auto-inhibiting ones, swinging active the sensitive Lck “mousetrap”. Indeed, the amount of Lck in the active open conformation was increased after ligand binding to the TCR [50,51]. 

Several mechanisms have been proposed to amplify or sustain TCR signals upon ligand binding once the phosphorylation of the ITAMs is initiated. For instance, ligand-induced receptor clustering, recruitment of the co-receptors CD4 and CD8, or the segregation of the TCR from phosphatases might be involved (reviewed in) [16]. In addition, the formation of a trimolecular complex between Lck, Nck and TSAd at the cytoplasmic tail of CD3ε might add to these amplifying mechanisms. It had been demonstrated that active Lck phosphorylates TSAd, which in turn establishes multiple interactions with Lck: (1) via the Lck(SH2) domain, and (2) between a PRS in TSAd and the Lck(SH3) domain [52]. These multiple interactions could trigger the recruitment or opening of additional Lck molecules or both, enhancing local Lck activity at the TCR [53]. Indeed, in our previous study, we have shown that Lck’s activity increases in the vicinity of the ligand-bound TCR [22].

Overall, in this study we proposed a model in which Lck and Nck cooperatively orchestrate optimal TCR signaling. First, ligand binding to the TCR stabilizes the active TCR conformation, exposing the cytoplasmic tail of CD3ε to its interaction partners, namely the PRS for the Nck(SH3.1) domain and the RK motif for the Lck(SH3) domain. In addition, the ITAMs are exposed to be phosphorylated by Lck. Binding of Nck to the PRS might help to stabilize the TCR in the active conformation or the binding of the Lck via its SH3 domain to the RK motif, or both. Of note, the latter interaction has been shown to be of low affinity compared to the interaction of the Nck(SH3.1) domain to the PRS [22,54], and therefore it might need some further Nck-mediated stabilization. Binding of the Lck(SH3) domain to the RK motif results in an increase in local Lck activity at the TCR, and consequently in ITAM phosphorylation. This increase might be mediated by three mechanisms: (1) by augmentation of the number of Lck molecules at the TCR due to Lck recruitment to CD3ε; (2) by Lck(SH3) domain intermolecular displacement via binding to the RK motif increasing the opening and activation of Lck; and (3) by further increasing local activity of Lck via opening of additional Lck molecules through interactions with TSAd or Nck or both, in conjugation with further amplification mechanisms. 

## Data Availability

Not applicable.

## Supplementary Materials

The following are available online at https://www.mdpi.com/article/10.3390/cells10040834/s1, Figure S1: Nck is needed for ZAP70 recruitment. Characterization of mock-treated control cells and shNck1/2 cells, and technical PLA controls between the TCR and ZAP70. Figure S2: Nck is required for Lck binding to the TCR. Binding of Nck(SH3.1) to the TCR assayed by Pull-Down assay with GST-Nck(SH3.1)-coupled beads, and technical PLA controls between the TCR and Lck. Figure S3: In vitro kinase assay of the TCR in the presence of Lck or Nck.
