# Supplementary figures and images for: Cooperative Interaction of Nck and Lck Orchestrates Optimal TCR Signaling
Source: Cells. 2021 Apr 7;10(4):834. doi: 10.3390/cells10040834 (PMC8068026; doi:10.3390/cells10040834)

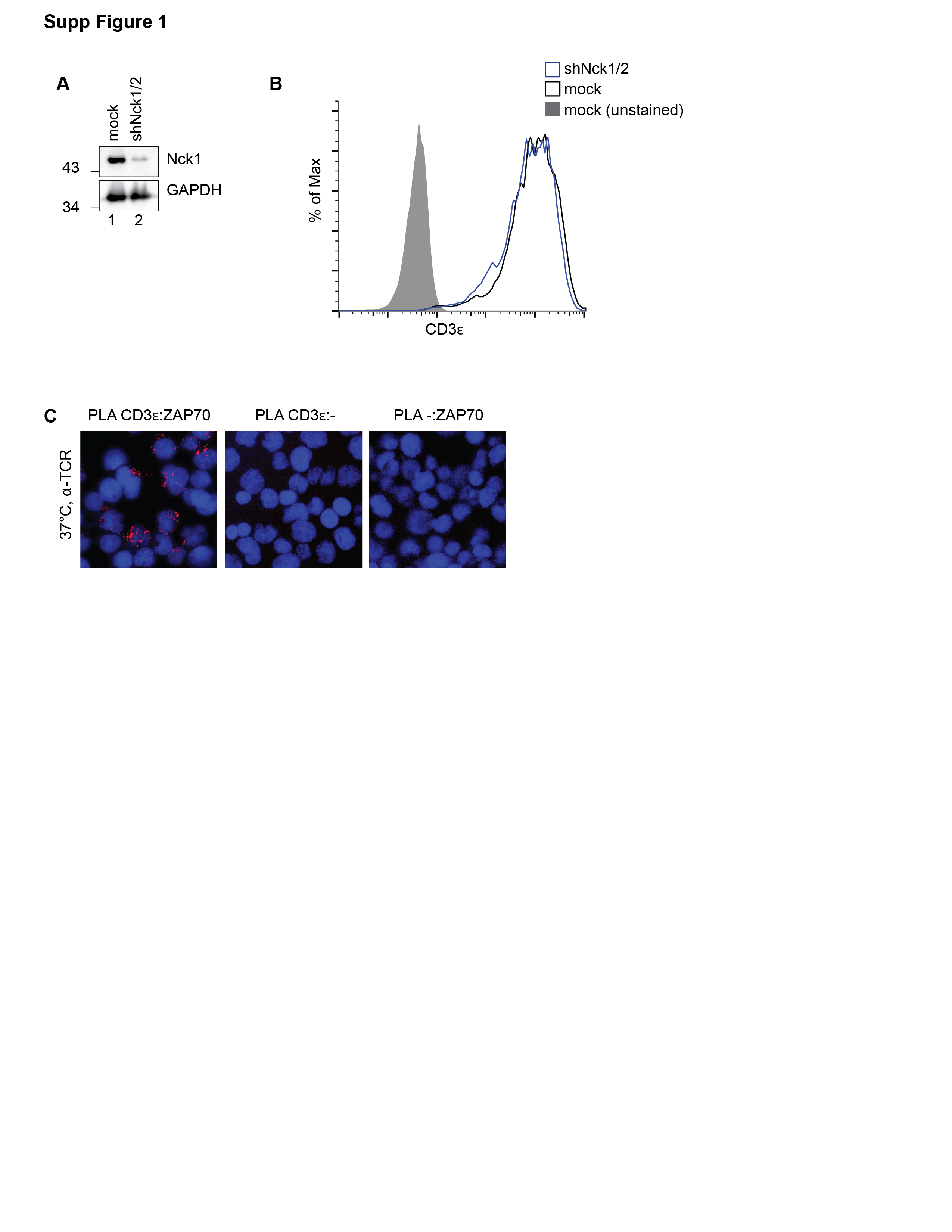

Supplement: Supplementary file 1 [file cells-10-00834-s001.zip › Hartl_Supp_Figure_1.png]

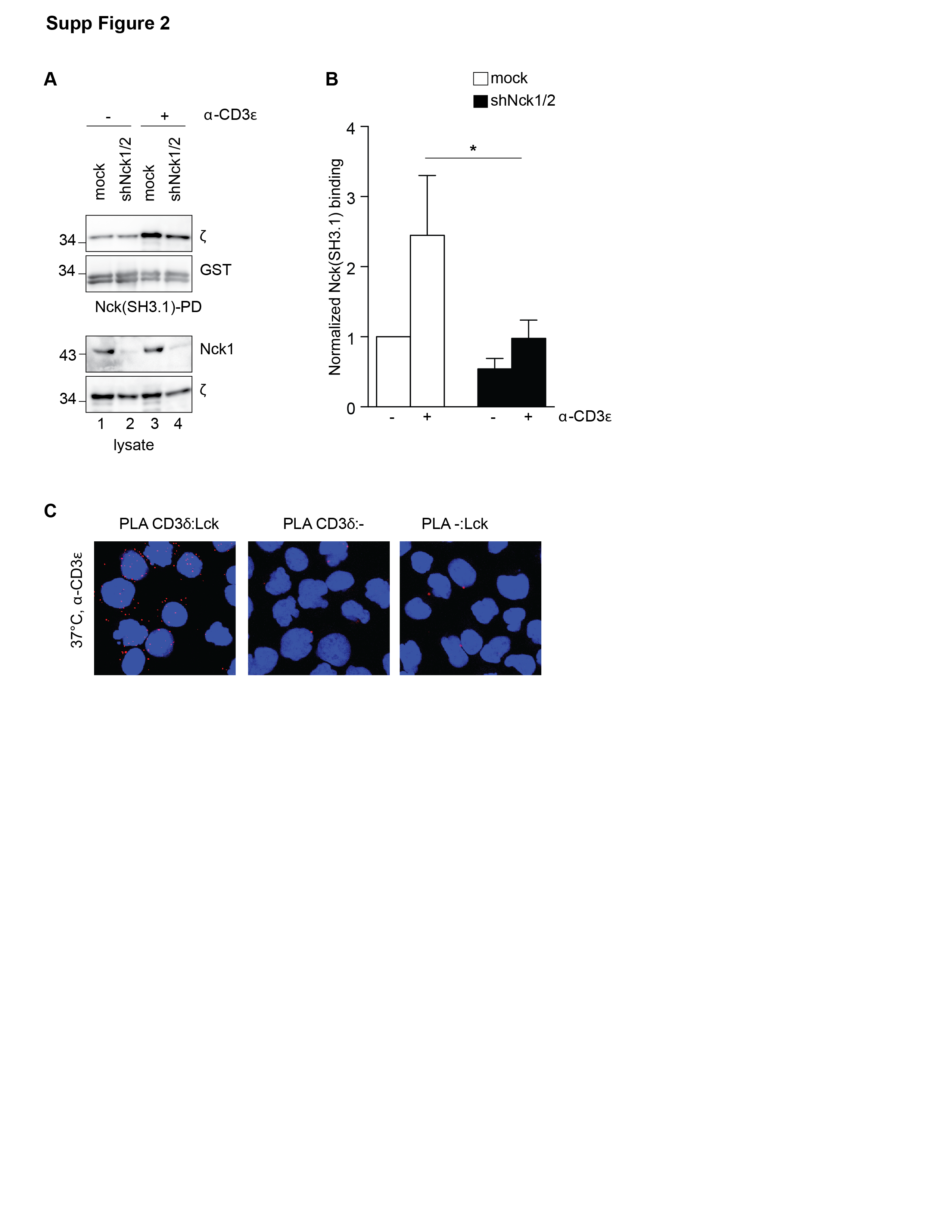

Supplement: Supplementary file 1 [file cells-10-00834-s001.zip › Hartl_Supp_Figure_2.png]

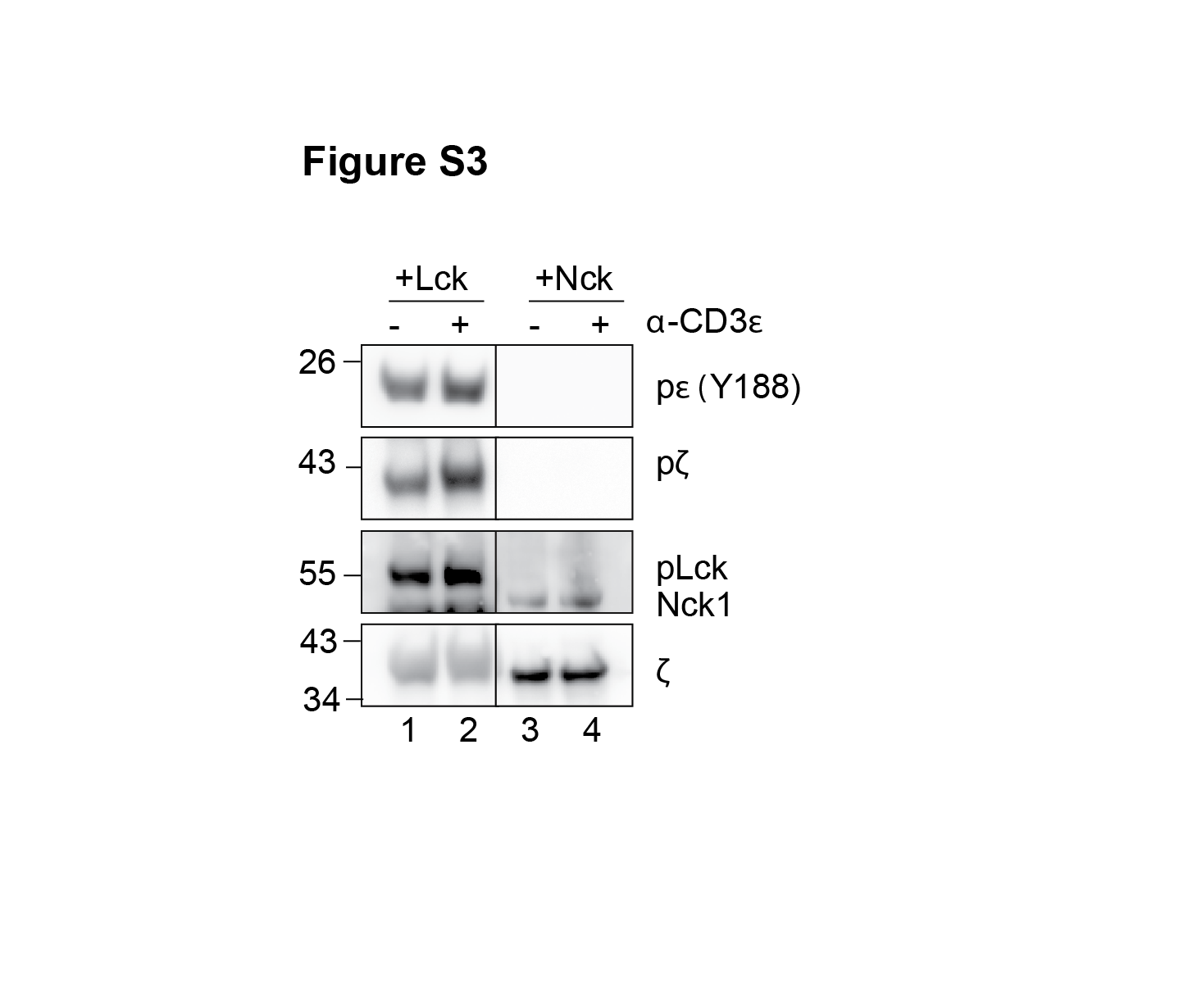

Supplement: Supplementary file 1 [file cells-10-00834-s001.zip › Hartl_Supp_Figure_3.png]
